# 

**Published:** 1889-05

**Authors:** 


### EDITORIAL.

### Dental Society Meetings.

The annual meeting of the Michigan State Society will be held in Grand Rapids, beginning on Tuesday, June 4th, 1889. An excellent programme, and indeed all provision, has been made for an interesting and successful meeting. The President, Dr. C. S. Case, and the Executive Committee have been untiring in their efforts to secure a good meeting, and from information just received there is no doubt as to the success. Grand Rapids is central, and withall a very attractive city. Preparation has been made for a large amount of clinic work, of every variety; and there will be on exhibition a large variety of new and improved appliances. The meeting will have such attractions as ought to draw to it every dentist in the State of Michigan; especially every one who has any interest in his profession.

While it is especially important that all the dentists of the State be present a hearty invitation is extended to all in the neighboring states to be present so far as practicable. Great good is accomplished by the interchange of visits by members of different state societies with each other: it stimulates friendly relationship, the value of which can not be easily estimated.

The annual meeting of the State Board of Dental Examiners will be held at the same time and place with the State Society. All parties interested should give special attention. It should be kept in mind that registration is required of all practitioners of dentistry in the State of Michigan; and also that all those desiring to enter practice without a diploma are required to pass a satisfactory examination before the Board. And in this examination practical demonstrations are required as well as answers given to written and oral questions. Michigan has a good law regulating dental practice and every one ought to conform strictly to its requirements. All enquiries pertaining to the work of the Board should be addressed to Dr. G. E. Corbin, Secretary, St. Johns, Michigan.

### Alumni Notice.

CHICAGO, April 2, 1889.

At the annual meeting of the alumni of the University of Michigan—Dental Department—held in Ann Arbor, June, 1888, a committee was appointed to take steps toward securing a large attendance at the next meeting in 1889, and in accordance with that intent, the committee beg your earnest co-operation, by your presence at the meeting, and by your sending the addresses of those alumni with whom you are personally acquainted, together with your own, to the above address. The committee ask that these addresses be forwarded at once, so that each alumnus may receive a personal invitation, for, owing to removal, many of the alumni have been lost sight of, and the list is far from perfect.

At the meeting, which will be held during commencement week in June, 1889, a special reunion of all classes previous to 1880 has been planned, and as soon as sufficient replies to this circular are received to warrant the formulating of special-class exercises, the committee will communicate with those signifying their intention of being present, so that it may be known who will be likely to attend. A varied programme of a scientific and social character will be prepared, and the exercises will close with a banquet.

An invitation is extended to classes graduated since 1880 to hold a reunion at the same time, and the committee promise to gladly assist in their organization. The alumni meeting occurring during the commencement week, will give those attending an opportunity to participate in the many social features of that occasion.

A prompt reply to this notice will lighten the labors of, and oblige, Yours sincerely,

L. L. Davis, (Class '84), Chr. of Com.

### THE DENTAL REGISTER,

A MONTHLY MAGAZINE DEVOTED TO THE INTERESTS OF THE

**DENTAL PROFESSION.**

*Terms Reduced to* $2.00 *Per Year. Single Copies,* 25 *Cents.*

**EDITED BY PROF. J. TAFT, D.D.S.**

—AND—

**Published by SAM'L A. CROCKER & CO., Ohio Dental and Surgical Depot.**

The volume commences in January and closes in December.

The REGISTER being issued monthly is always fresh, therefore Indispensable to the practitioner who desires to keep posted up with the new inventions and improvements which are daily developed. Neither expense nor effort will be spared to give value and variety to its contents. Articles will be illustrated with well executed engravings whenever they will add to the interest or assist to explanation.

Its extensive circulation and frequency of issue make it one of the BEST DENTAL ADVERTISING MEDIUMS in the United States.

All subscriptions must begin with January or July.

Local or special notices, five lines or less, will be inserted for $3 each insertion; over five lines, 50 cts. per line.

All advertising bills payable in advance, or at furthest, after the issue of the first number containing the advertisement. All transient advertisements to be paid for in advance.

☞CONTRIBUTIONS SOLICITED.

Exclusively Editorial Communications should be addressed to the Editor.

The latest number of the REGISTER may be ordered with or without other goods at any time, and all letters should be addressed to

SAM'L A. CROCKER & CO., Ohio Dental and Surgical Depot, Cincinnati, O.

